# Supplementary material for: Hardware implementation of Bayesian network based on two-dimensional memtransistors
Source: Nat Commun. 2022 Sep 23;13:5578. doi: 10.1038/s41467-022-33053-x (PMC9508127; doi:10.1038/s41467-022-33053-x)
Supplement: Supplementary file 1 — Supplementary Information [file 41467_2022_33053_MOESM1_ESM.pdf]

Supplementary Information

Hardware Implementation of Bayesian Network based on Two-dimensional Memtransistors

Yikai Zheng[1], Harikrishnan Ravichandran[1], Thomas F Schranghamer[1], Nicholas Trainor[2,3], Joan M Redwing[2,3], and Saptarshi Das[1,2,3,4,]*

[1]Engineering Science and Mechanics, Penn State University, University Park, PA 16802, USA

[2]Materials Science and Engineering, Penn State University, University Park, PA 16802, USA

[3]Materials Research Institute, Penn State University, University Park, PA 16802, USA

[4]Electrical Engineering and Computer Science, Penn State University, University Park, PA 16802, USA

Corresponding author email: sud70@psu.edu

Supplementary Figure 1

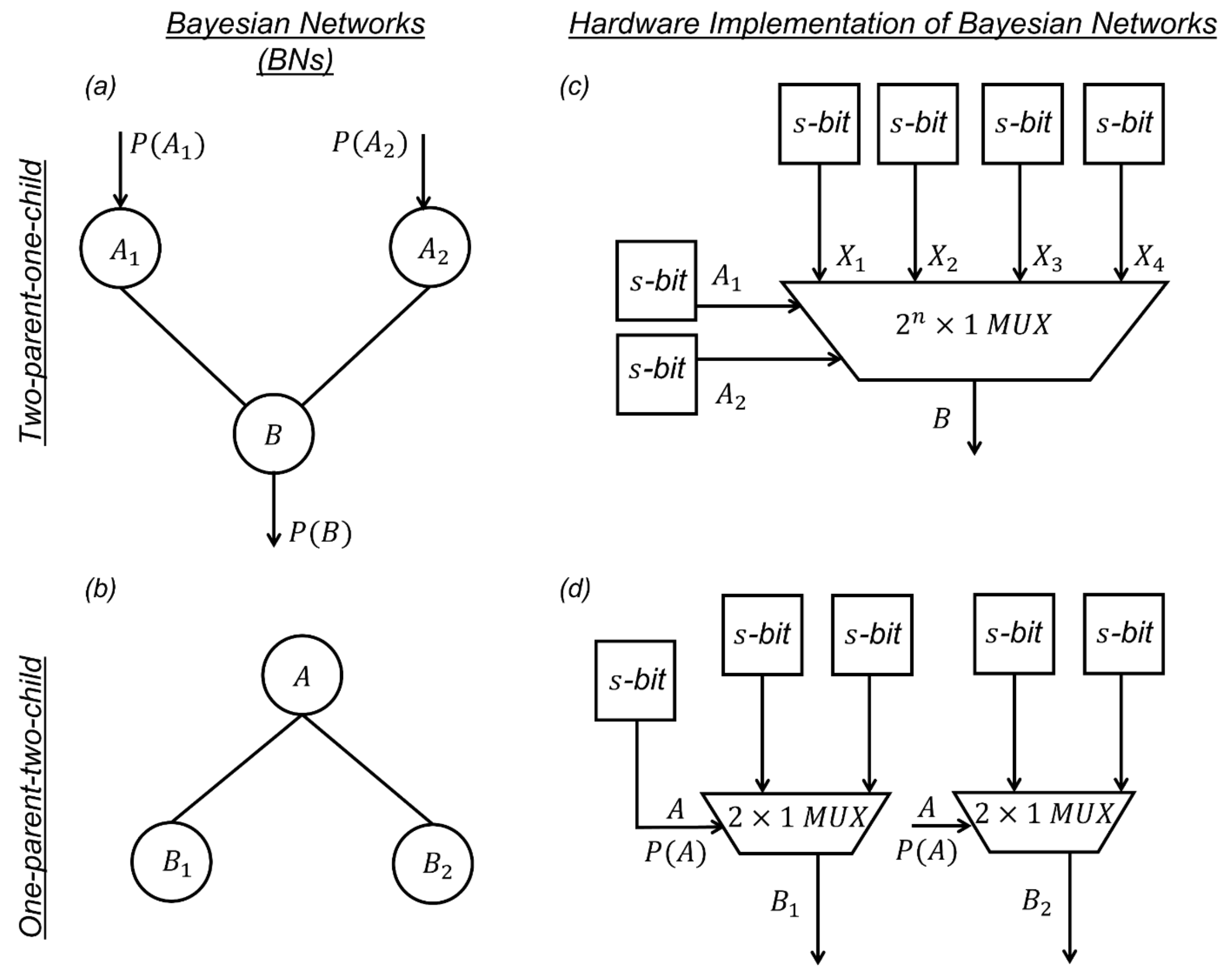

Figure S1. Example Bayesian networks (BN) and their hardware implementations. a) A BN where the child node, B is connected to 2 parent nodes, A_1 and A_2. b) A BN where the parent node, A is connected to 2 children, B_1 and B_2. c) Hardware implementation of BN shown in (a) can be achieved by using 2 s-bit generators to obtain the A_1 and A_2. Another N = 4 s-bit generators to obtain the CPT, and one 4 × 1 MUX with 2 select lines. d) Hardware implementation of BN shown in (b) can be achieved by using 1 s-bit generator to obtain A. Another 4 s-bit generators to obtain the 2 CPTs, and 2

Supplementary Figure 2

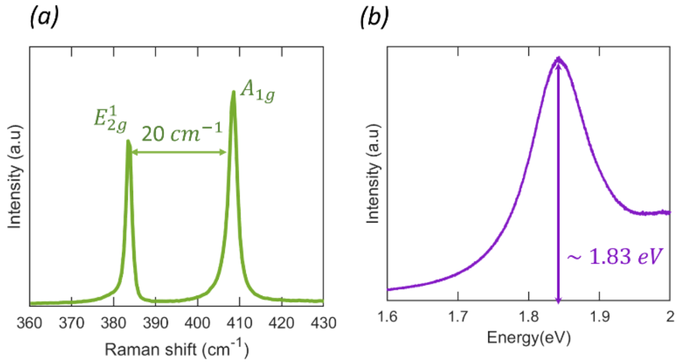

Figure S2. Monolayer MoS_2 characterization. a) Raman spectra obtained for a representative 2D memtransistor shows two monolayer MoS_2 peaks at 383 cm^{-1} and 404 cm^{-1} corresponding to the in-plane E_{2g} and out-of-plane A_{1g} modes, respectively, with an expected peak separation of ~20 cm^{-1}. b) Photoluminescence (PL) spectra for a representative 2D memtransistor shows a peak at 1.83 eV corresponding to the direct bandgap of monolayer MoS_2.

Supplementary Figure 3

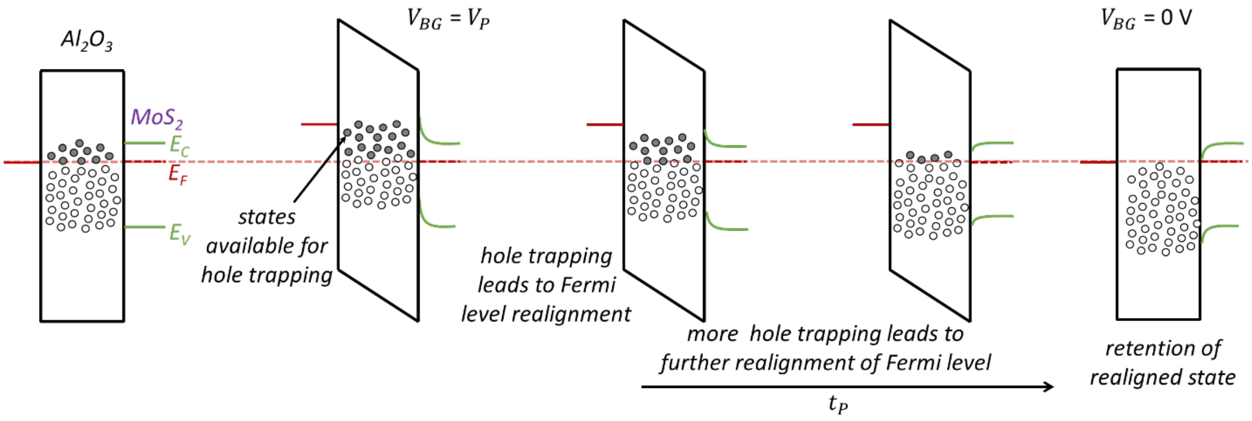

Figure S3. Energy band diagram. Energy band diagram explaining the charge trapping phenomena while programming the 2D memtransistor.

Supplementary Figure 4

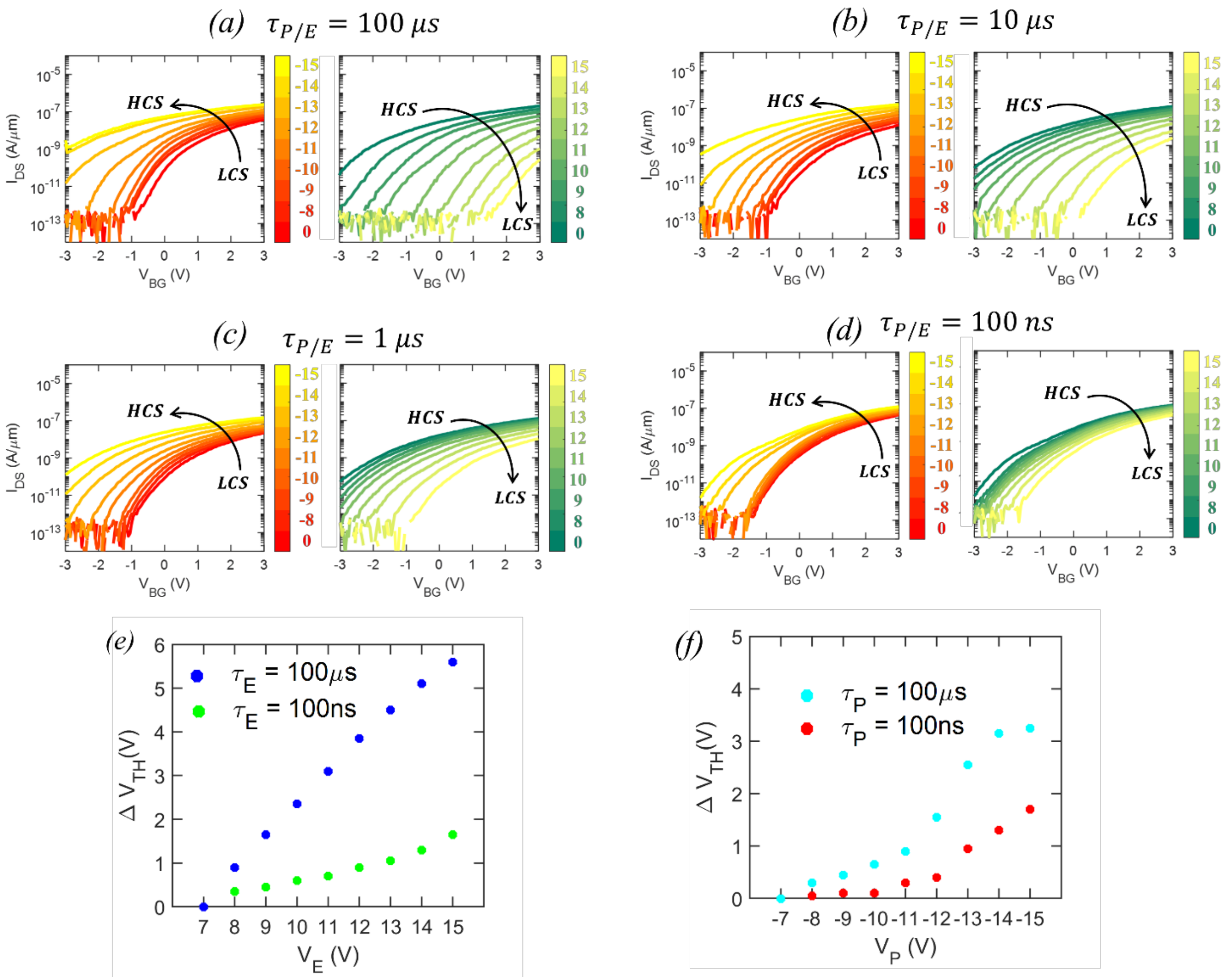

Figure S4. Effect of programming pulse duration on the performance of MoS_2 memtransistor. Post-programmed and Post-erased transfer characteristics of a 2D memtransistor subjected to negative "Write" (V_P) and positive "Erase" (V_E) voltage pulses of different amplitudes ranging from 8 V to 15 V applied to the local back-gate electrode, each for a duration of a) $\tau_{P/E}$ = 100 µs, b) $\tau_{P/E}$ = 10 µs, c) $\tau_{P/E}$ = 1 µs, and d) $\tau_{P/E}$ = 100 ns. Extracted shift in the threshold voltage (ΔV_{TH}) as a function of $V_{P/E}$ for e) $\tau_{P/E}$ = 100 µs and f) $\tau_{P/E}$ =100 ns.

Supplementary Figure 5

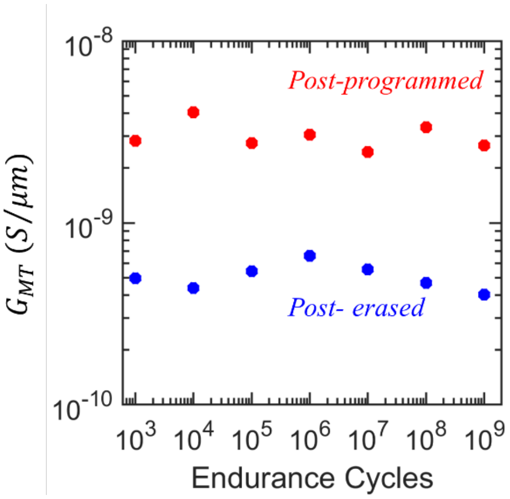

Figure S5. Endurance data for MoS_2 memtransistor. Programming endurance for 2D memtransistor, taken using V_P = -7 V and V_E = 10 V with $\tau_{P/E}$ = 100 ns for over 109 cycles.

Supplementary Figure 6

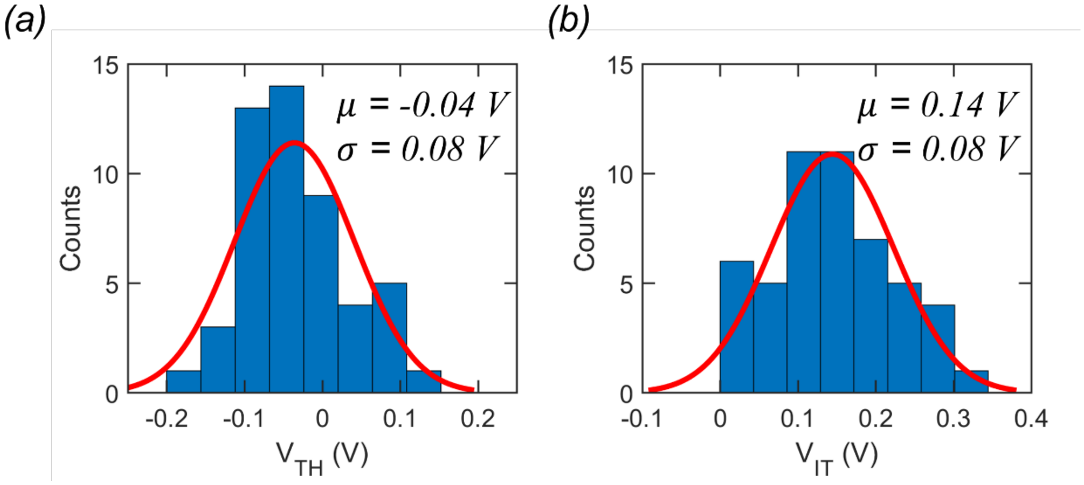

Figure S6. Cycle-to-cycle programming variation. a) Distribution of cycle-to-cycle programming variation in the V_{TH} of MT_6 and b) the corresponding variation in the V_{IT} of the thresholding inverter when MT_6 is subjected to 50 program/erase/read cycles with V_P = -7V, V_E = 10 V and $\tau_{P/E}$ = 100 µs.

Supplementary Figure 7

**Table 1. NIST Test on s-bit streans**		
NIST Test	*p-value*	*Result*
Frequency Monobit Test	*0.0357*	*Pass*
Frequency Test within a Block	*0.3933*	*Pass*
Runs Test	*0.1433*	*Pass*
Longest Run of Ones	*0.0935*	*Pass*
Binary Matrix Rank Test	*0.3230*	*Pass*
Discrete Fourier Transform Test	*0.0669*	*Pass*
Serial Test	*0.1561*	*Pass*
Cumulative Sums (Cusum) Test	*0.3673*	*Pass*

Figure S7. NIST test results. Results of NIST tests for s-bits.

Supplementary Figure 8

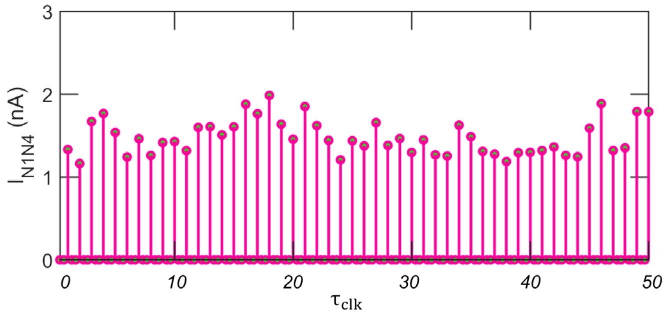

Figure S8. Total current for the s-bit generation. Current measured between N_1 and N_4, i.e., I_{N1N4}, for 50 clock cycles.

Supplementary Figure 9

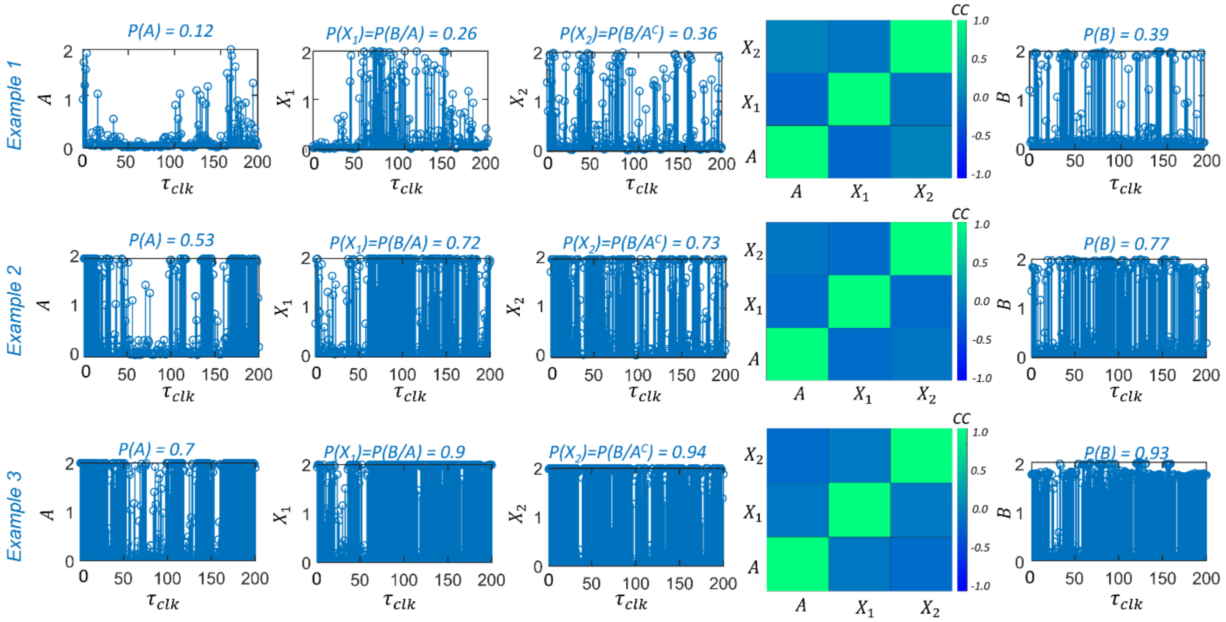

Figure S9. Examples of output from the BN for different input bit-streams. Three examples of representative stochastic bit-streams for the random variables A, X_1, and X_2, correlation coefficient (CC) values between A, X_1, and X_2, and the stochastic bit-streams obtained at the output node, B for the 2-node BN. The measured and expected values for $P(B)$ are very similar confirming high precision hardware implementation of the BN.

Supplementary Figure 10

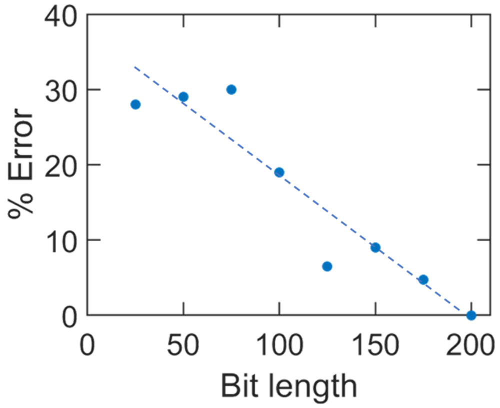

Figure S10. Error in the output of the BN for different bit-lengths. Percentage error of $P(B)$ as a function of bit length of s-bit streams, with the expected value of the BN output, $P(B) = 0.54$, with $P(A) = 0.59$, $P(B/A) = 0.39$, and $P(B/A^C) = 0.75$.

Supplementary Figure 11

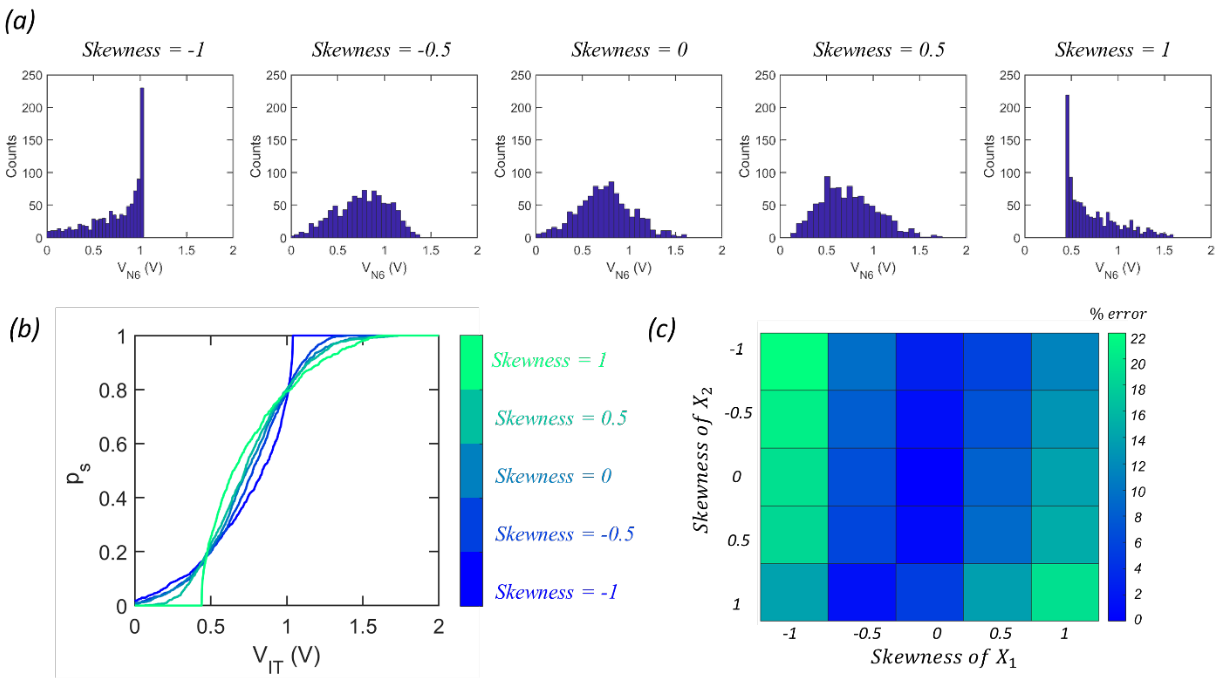

Figure S11. Simulation results showing the impact of skewness on the BN. a) Simulation results showing V_{N6}, i.e., the output of the inverting amplifier of the s-bit generator circuit drawn from Pearson random distribution function with different skewness. b) Corresponding p_s as a function of V_{IT}. As the skewness increases, the deviation of p_s from its expected value also increases. c) Colormap of the percentage error in estimating $P(B)$ using the hardware BN architecture for different skewness in the stochastic input variables X_1 and X_2 that represent $P(B/A)$ and $P(B/A^C)$, respectively.

Supplementary Figure 12

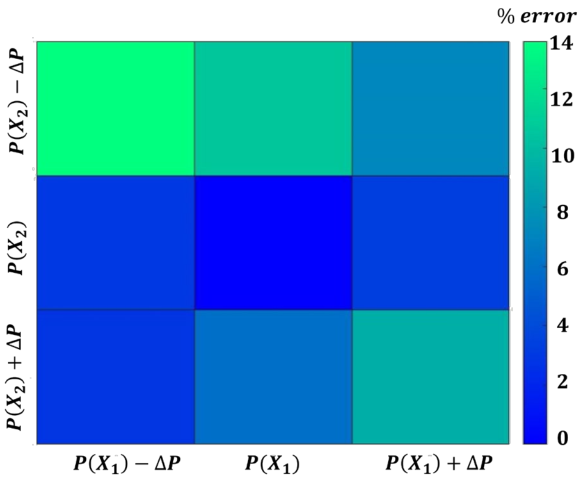

Figure S12. Simulation results showing the impact of cycle-to-cycle variation in the threshold voltage on the output of BN. $P(B)$ for the 2-node BN ($B = AX_1 + A^C X_2$) due to cycle-to-cycle variation of V_{IT}, of the thresholding inverter (Supplementary Fig. S6b) leading to uncertainty ($\Delta P \approx 0.065$) in the stochastic input values $P(X_1)$ and $P(X_2)$. $P(X_1)$ represents $P(B/A) = 0.50$, and $P(X_2)$ represents $P(B/A^C) = 0.56$ while the select line, $P(A) = 0.28$, remains constant.

Supplementary Figure 13

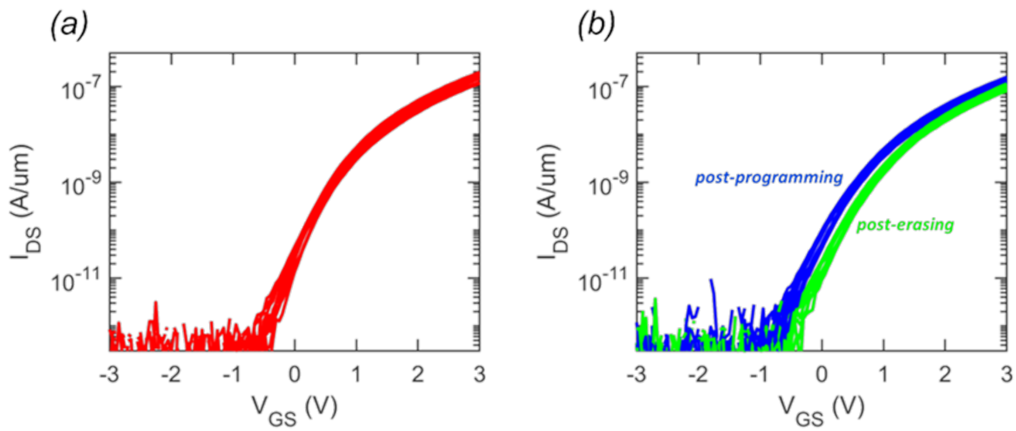

Figure S13. Device-to-device variation in MoS_2 memtransistors. (a) Transfer characteristics of 10 MoS2 memtransistors. (b) Transfer characteristics of the 10 MoS2 memtransistors after subjecting to programming/erasing voltage pules ($V_P = -7V$, $V_E = 10V$) each for a duration of τ_s = 100µs.

Supplementary Figure 14

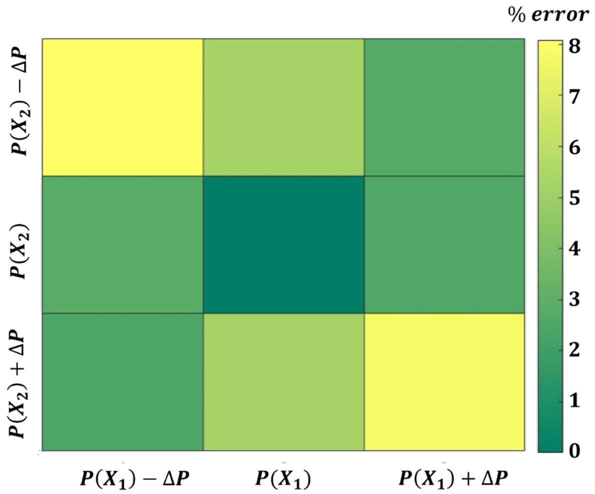

Figure S14. Simulation results showing the impact of device-to-device variation on BN output. $P(B)$ for the 2-node BN ($B = AX_1 + A^C X_2$) due to device-to-device variation (Supplementary Fig. S13b) leading to uncertainty ($\Delta P \approx 0.046$) in the stochastic input values $P(X_1)$ and $P(X_2)$. $P(X_1)$ represents $P(B/A) = 0.50$, and $P(X_2)$ represents $P(B/A^C) = 0.56$, while the select line, $P(A) = 0.28$, remains constant.
